# Investigation of the Hydrolysis of Perovskite Organometallic Halide CH_3_NH_3_PbI_3_ in Humidity Environment

**DOI:** 10.1038/srep21976

**Published:** 2016-02-29

**Authors:** Jiangtao Zhao, Bing Cai, Zhenlin Luo, Yongqi Dong, Yi Zhang, Han Xu, Bin Hong, Yuanjun Yang, Liangbin Li, Wenhua Zhang, Chen Gao

**Affiliations:** 1 National Synchrotron Radiation Laboratory and CAS Key Laboratory of Materials for Energy Conversion, University of Science and Technology of China, Hefei, Anhui, 230026, China; 2 State Key Laboratory of Catalysis, Dalian Institute of Chemical Physics, Chinese Academy of Sciences, Dalian National Laboratory for Clean Energy, Dalian 116023, China

## Abstract

Instability of emerging perovskite organometallic halide in humidity environment is the biggest obstacle for its potential applications in solar energy harvest and electroluminescent display. Understanding the detailed decay mechanism of these materials in moisture is a critical step towards the final appropriate solutions. As a model study presented in this work, *in situ* synchrotron radiation x-ray diffraction was combined with microscopy and gravimetric analysis to study the degradation process of CH_3_NH_3_PbI_3_ in moisture, and the results reveal that: 1) intermediate monohydrated CH_3_NH_3_PbI_3_·H_2_O is detected in the degradation process of CH_3_NH_3_PbI_3_ and the final decomposition products are PbI_2_ and aqueous CH_3_NH_3_I; 2) the aqueous CH_3_NH_3_I could hardly further decompose into volatile CH_3_NH_2_, HI or I_2_; 3) the moisture disintegrate CH_3_NH_3_PbI_3_ and then alter the distribution of the decomposition products, which leads to an incompletely-reversible reaction of CH_3_NH_3_PbI_3_ hydrolysis and degrades the photoelectric properties. These findings further elucidate the picture of hydrolysis process of perovskite organometallic halide in humidity environment.

Nowadays, solar cells which use perovskite organometallic halide as light absorption layer have aroused a vast concern all over the world, due to the merits of high efficiency, low-cost and simple synthesis process of the perovskite materials^1^,^2^,^3^,^4^,^5^,^6^,^7^,^8^,^9^,^10^,^11^,^12^. In 2009, power conversion efficiency (PCE) of 3.81% was firstly demonstrated in perovskite solar cell with spin-coated CH_3_NH_3_PbI_3_ on FTO^1^. Six years later, PCE as high as ~20% has been acquired through continuous efforts in controlling the formation of the perovskite layer and choosing appropriate material for other layers^2^,^3^,^4^,^5^,^6^,^7^. The quickly enhanced PCE seems signal a new era of perovskite solar cells.

Unfortunately, the instability of organometallic halide perovskite in humidity environment is a tough problem, which hinders its practical application in solar cells and electroluminescent display. Therefore, it is urgent to make clear the decay mechanism of this easily air-slaked material, which is fundamentally meaningful and is also a critical step for pursuing more appropriate solutions. As a model system of perovskite organometallic halide, CH_3_NH_3_PbI_3_ has been intensively studied recently, including the instability problem in moisture^13^,^14^,^15^,^16^,^17^,^18^,^19^,^20^. For instance, in 2014, Niu and Frost *et al* proposed a set of degradation equations^16^ and a possible decomposition pathway^17^, respectively. Thereafter, more experimental evidences were reported by Yang^18^, Christians^19^ and Leguy^20^ *et al.* Yang and co-workers utilized *in situ* grazing incidence X-ray diffraction to monitor the phase evolution of CH_3_NH_3_PbI_3_ in water vapor, where an intermediate phase was found and supposed to be (CH_3_NH_3_)_4_PbI_6_ • 2H_2_O^18^. This intermediate phase was also discovered by Christians *et al*^19^. Moreover, Leguy *et al* reported a more systematic work performed with time-resolved XRD and ellipsometry, in which two different hydrated crystalline phases of CH_3_NH_3_PbI_3_, i.e., CH_3_NH_3_PbI_3_ • H_2_O and (CH_3_NH_3_)_4_PbI_6_ • 2H_2_O were detected and a suite of convincing degradation equations were provided^20^. However, despite all these, more detailed questions should be answered, like ‘what are the real final decomposition products?’ and ‘what is the function of moisture in the degradation process?’.

To address these issues, here, we carefully studied the degradation process of typical perovskite organometallic halide CH_3_NH_3_PbI_3_ in moisture. Using *in-situ* synchrotron XRD experiment, four distinct states have been revealed and in which monohydrated CH_3_NH_3_PbI_3_ • H_2_O and one decomposition product PbI_2_ are found. Another decomposition product, CH_3_NH_3_I, is confirmed through a solid-liquid separation experiment and it was demonstrated to be difficult for aqueous CH_3_NH_3_I to transform to volatile matter through a gravimetric analysis. The microscopy analysis reveals that the reaction of CH_3_NH_3_PbI_3_ with water vapor is not a completely reversible one, because the moisture alters the distribution of decomposition products and thus partially separates PbI_2_ and CH_3_NH_3_I.

## Results and Discussion

### *In situ* synchrotron radiation XRD experiment

**Figure 1:**
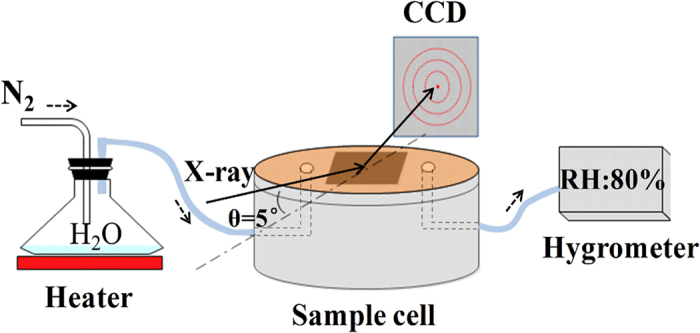
Schematic diagram of the RH control device and the diffraction geometry in the *in-situ* XRD experiment.

Figure 1 is the schematic of the experiment setup, in which CH_3_NH_3_PbI_3_/FTO film was placed in a homemade sample cell at the diffractometer center. The relative humidity (RH) in the cell was controlled by tuning the flow rate of nitrogen gas and the temperature of the heater. The RH value was real-time monitored by a commercial hygrometer and which was regulated to about 80% during the whole experiment. To confine the moisture, the cell was sealed with kapton film which will not block the incident and diffractive x-ray beam passing through.

**Figure 2:**
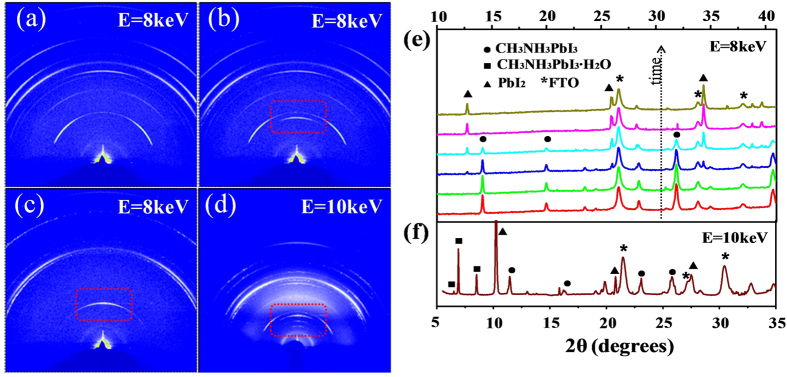
*In situ* XRD patterns. (**a–d**) Typical diffraction patterns of spin-coated CH_3_NH_3_PbI_3_/FTO in degradation process and (**e–f**) the corresponding integral curves obtained by using Fit2D program.

Figure 2 shows the typical diffraction patterns selected from the large set of data collected during the *in situ* synchrotron radiation XRD experiment, which indicates the degradation process of spin-coated CH_3_NH_3_PbI_3_/FTO in moisture.

Figure 2a–c are three typical patterns taken during one test (X-ray photon energy E = 8keV) with a time sequence of a → b → c, and the corresponding integrated curves are presented in Fig. 2e among those belong to the time-serial data set. Figure 2a is the diffraction pattern of the as-grown CH_3_NH_3_PbI_3_/FTO without water vapor around the sample, which reveals that the film is polycrystalline with preferred orientation along the normal direction. As shown in Fig. 2b, with the time increasing in a relative humidity of 80 ± 5%, the diffraction peaks of perovskite CH_3_NH_3_PbI_3_ gradually decrease due to degradation and the reflections belonging to PbI_2_ (PDF#07-0235) become more and more intense. Figure 2c shows that the final decomposition product is composed mainly by PbI_2_. However, according to the law of conservation of elements, at least another decomposition product should be there. Since no obvious peaks from other phases appear in this pattern, that product is believed to be volatilized or dissolved in water. As described in detail in the following part, the product is proved to be aqueous CH_3_NH_3_I.

When we repeated the *in situ* XRD measurements, sometimes, we could fortunately detect a metastable phase during the degradation process. One of such patterns is presented in Fig. 2d and the integral curve is plotted in Fig. 2f. The peaks at 6.5°, 6.9° and 8.5° are ascribed to the (001), (110) and (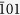) reflections of the metastable monoclinic CH_3_NH_3_PbI_3_·H_2_O^20^. Please note that such intermediate phases were not obvious every time during our XRD experiments, because it is difficult to catch these time- and space-limited phases without enough diffraction intensity by using x-ray beam with sub-millimeter size. Of course, it is more possible to detect such hydrates if people delay the degradation process by decreasing the relative humidity around the sample.

#### Microscopy Analysis

**Figure 3:**
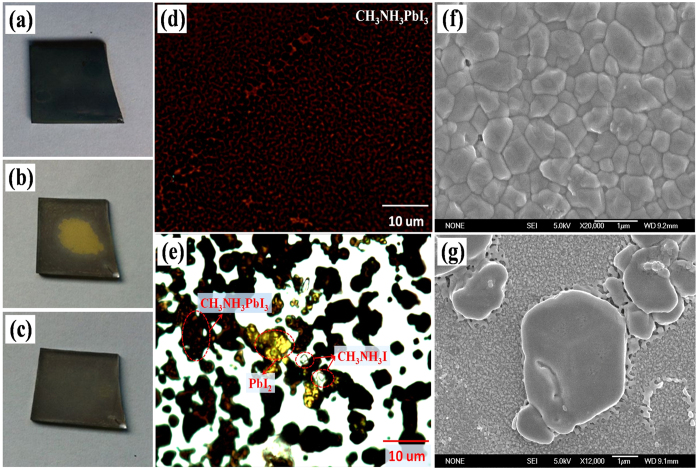
Microscopy images of CH_3_NH_3_PbI_3_/FTO film before and after decaying. (**a**) Photographs of the as-grown CH_3_NH_3_PbI_3_/FTO film and (**b,c**) the decayed CH_3_NH_3_PbI_3_ films after taken out from the moisture. (**d**) Optical micrographs of the as-grown CH_3_NH_3_PbI_3_/FTO film and (**e**) the decayed CH_3_NH_3_PbI_3_ film. (**f**) SEM images of the as-grown CH_3_NH_3_PbI_3_/FTO film and (**g**) the decayed CH_3_NH_3_PbI_3_ film.

During the *in situ* XRD experiment, the as-grown brown black CH_3_NH_3_PbI_3_/FTO film (Fig. 3a) gradually becomes yellow (Fig. 3b) in the humidity environment. But, it is interesting that the surface of the film slowly turns yellow to light black (Fig. 3c) after we take the film out of the humidity condition, which seems should be attributed to the water evaporating from the sample.

This phenomenon combined with XRD results (data not show here) indicates the reaction between CH_3_NH_3_PbI_3_ and water vapor is not fully reversible. We guess the underlying cause is that the water vapor not only decompose CH_3_NH_3_PbI_3_ into PbI_2_ and aqueous CH_3_NH_3_I, but also separates these products to some degree. To demonstrate this hypothesis, optic microscopy and scanning electron microscopy (SEM) were utilized to investigate the film morphology before and after the degradation.

Figure 3d is a typical optical micrograph of the as-grown spin-coated CH_3_NH_3_PbI_3_/FTO film. The uniform brown black color indicates the film is homogeneous without obvious second phase. Similar distribution state is also revealed from the corresponding SEM image (Fig. 3f). For the film after the degradation, three types of grain with different color could be easily recognized from the optical micrograph Fig. 3e. The yellow particles are PbI_2_, bright transparent grains should be CH_3_NH_3_I, and the brown black ones are the revived CH_3_NH_3_PbI_3_. In addition, the distribution of these color grains were found in an obvious isolated and disorder manner in comparison with the initial CH_3_NH_3_PbI_3_ film. The SEM image in Fig. 3g also clearly illustrates the rough morphology of the decayed CH_3_NH_3_PbI_3_ film. The above microscopy analysis proves that the moisture indeed alters the distribution of decomposition products and thus makes the reverse reaction between CH_3_NH_3_PbI_3_ and water insufficient.

Till now, the degradation process and decay mechanism may be expressed as follows:


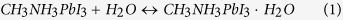



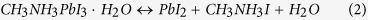


#### Solid-Liquid Separation Methods and Gravimetric Analysis

As shown in the microscope images, the separation of PbI_2_ and CH_3_NH_3_I in real space is the key step towards the irreversible reaction between CH_3_NH_3_PbI_3_ and water. The cause of this separation is thought to be the different solubility of PbI_2_ and CH_3_NH_3_I in water, which is verified by the following solid-liquid separation experiment.

**Figure 4:**
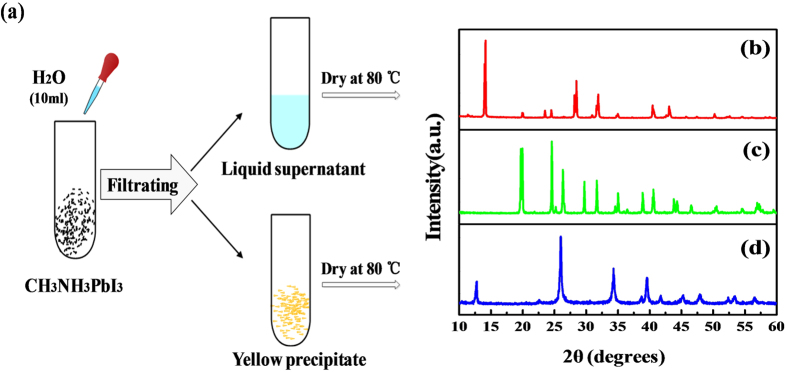
(**a**) Schematic diagram of solid-liquid separation experiment; XRD patterns of (**b**) CH3NH3PbI3 powder, (**c**) the resulting powder separated out from the liquid supernatant and (**d**) dried yellow precipitate.

Here, CH_3_NH_3_PbI_3_ powder is used as the research object to make things simple and clear. The steps of the experiment are schematically shown in Fig. 4a. First, CH_3_NH_3_PbI_3_ powder (XRD pattern presented in Fig. 4b) was put in distilled water, and the black powder was found turned into yellow precipitate at once. Then, the liquid supernatant and the yellow precipitate were separated by centrifuging and filtrating. Both the liquid supernatant and yellow precipitate were dried at 80  °C in dark and the resulting powders were characterized by XRD (Fig. 4c,d), respectively.

From the XRD patterns, it is found that the soluble product of CH_3_NH_3_PbI_3_ hydrolysis is CH_3_NH_3_I (PDF#10-0737) and the yellow precipitate is PbI_2_ (PDF#07-0235). The difference in solubility of these two products is the major reason for separation.

**Figure 5:**
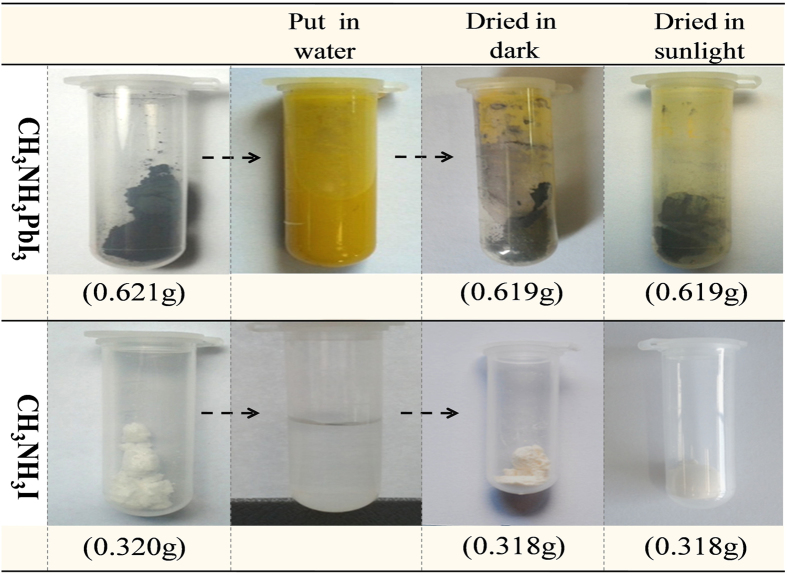
Color and weight change of CH_3_NH_3_PbI_3_ (1^st^ row) and CH_3_NH_3_I (2^nd^ row) powder when dried in dark or sunlight.

Back to the aforementioned another question, i.e. ‘what are the real final decomposition products of CH_3_NH_3_PbI_3_ hydrolysis’, previous studies mostly claim that the final rest product is PbI_2_ while the aqueous CH_3_NH_3_I will volatilize as CH_3_NH_2_, HI or I_2_ in the condition of moisture and sunlight. Detailed information can be found in ref.^16^,^17^,^21^. Noted that CH_3_NH_3_PbI_3_/TiO_2_/FTO hetero-structure was used in these studies, it could not exclude the interfacial effect^21^ in CH_3_NH_3_PbI_3_/TiO_2_ since TiO_2_ is well-known photocatalytic material^22^. Therefore, another question emerged naturally is what the hydrolysis products of stand-alone CH_3_NH_3_PbI_3_. Here, a high-precision gravimetric analysis has been carried out to investigate whether the aqueous CH_3_NH_3_I will further volatilize for CH_3_NH_3_PbI_3_ powders, and the results are listed in Fig. 5. First, two sets of 0.621g CH_3_NH_3_PbI_3_ powder and two sets of 0.320g CH_3_NH_3_I powder were put in test tubes separately, and then 5 ml distilled water was injected in each test tube. Next, one set of watered CH_3_NH_3_PbI_3_ and one set of aqueous CH_3_NH_3_I were directly dried in dark, i.e. in the drying oven at 80  °C for a week, while the rest were dried in sunlight for the same time. Finally, all the dried powders were weighted using electronic balance with a precision of 0.01 mg.

The 1^st^ row in Fig. 5 reveals the color and weight change of CH_3_NH_3_PbI_3_ powder in this experiment. Notice that the CH_3_NH_3_PbI_3_ decayed in water changes back to black again either dried in dark or in sunlight, which confirm that the reaction between CH_3_NH_3_PbI_3_ and water is almost reversible if the decomposition products are not separated. As shown by the weight values listed in the figure, the CH_3_NH_3_PbI_3_ powder almost does not loss any weight either dried in dark or in sunlight, which indicates that no obvious volatile matter release during CH_3_NH_3_PbI_3_ hydrolysis, i.e. it is hard for CH_3_NH_3_I to further separate. The conclusion was also verified by the control experiment performed on CH_3_NH_3_I powder, in which the results listed in the 2^nd^ row show that the CH_3_NH_3_I powder weight almost does not reduce in any cases. In the above measurements, no reflections from other phases were detected by XRD before and after gravimetric analysis (data not shown here). Please note that, the results and conclusion presented here are much different from that found in CH_3_NH_3_PbI_3_/TiO_2_/FTO hetero-structure, indicating TiO_2_ could influence the degradation process of CH_3_NH_3_PbI_3_.

Now, it could be said that the above decay equations (1 and 2) are the major reaction for stand-alone CH_3_NH_3_PbI_3_, and the electric transport layer (ETL) in perovskite solar cells may affect the stability of perovskite organometallic hilides^23^,^24^,^25^,^26^. Our findings in this paper further elucidate the picture of hydrolysis process of this perovskite material in humidity environment.

## Conclusion

Based on *in situ* synchrotron radiation XRD, a reasonable decomposition pathway for CH_3_NH_3_PbI_3_ materials in moisture is proposed and the intermediate monohydrated CH_3_NH_3_PbI_3_·H_2_O is detected during the degradation process. Moreover, the function of moisture was further investigated via microscopy and gravimetric analysis. The results show that moisture not only decompose CH_3_NH_3_PbI_3_ into PbI_2_ and aqueous CH_3_NH_3_I but also altered their distribution, which result in poor connection between PbI_2_ and CH_3_NH_3_I and thus leads to an incompletely-reversible reaction of CH_3_NH_3_PbI_3_ hydrolysis. Furthermore, it is confirmed that the final products of CH_3_NH_3_PbI_3_ hydrolysis are just PbI_2_ and aqueous CH_3_NH_3_I, and CH_3_NH_3_I will hardly further decompose into volatile CH_3_NH_2_, HI or I_2_ for CH_3_NH_3_PbI_3_ itself. The degradation process and decay mechanism of perovskite CH_3_NH_3_PbI_3_ in moisture becomes clearer with this work, which is believed to be helpful to understand the instability problem in moisture of various perovskite organometallic halides and figure out the possible solutions according to their applications.

## Methods

To prepare the CH_3_NH_3_PbI_3_ specimens, equimolar CH_3_NH_3_I and PbI_2_ (98%, Sinopharm) were mixed in γ-butyrolactone (97%, Sinopharm) at 60  °C overnight whilst being stirred. Then, the aqueous precursor was deposited on FTO glass by spin-coating at 2000 rpm for 40s. Finally, the specimens were annealed at 100  °C for 15 min on a preheated hot plate, and the obtained CH_3_NH_3_PbI_3_/FTO films were brown black^27^.

*In situ* synchrotron radiation XRD was performed at the BL14B station of Shanghai Synchrotron Radiation Facility (SSRF), and the experimental setup is schematically shown in Fig. 1. The diffractive patterns were recorded by a two-dimension x-ray detector (charge coupled device, CCD) with 3072 × 3072 pixels. To monitor the reaction at real-time, the diffraction patterns were continuously recorded with 10 seconds per frame and 1 second between adjacent frames.

To investigate the CH_3_NH_3_PbI_3_ film morphology before/after the reaction, microscopy analysis was performed by using optical microscope (OLYMPUS, BX51) and scanning electron microscope (SEM, JSM-6700F).

## Additional Information

**How to cite this article**: Zhao, J. *et al.* Investigation of the Hydrolysis of Perovskite Organometallic Halide CH3NH3PbI3 in Humidity Environment. *Sci. Rep.* **6**, 21976; doi: 10.1038/srep21976 (2016).

## References

[b1] Kokima A., Teshima K., Shirai Y. & Miyasaka. Organometal halide perovskites as visible-light sensitizers for photovoltaic cells. J. Am. Chem. Soc. 131, 6050–6051 (2009). 19366264 10.1021/ja809598r

[b2] Im J. H., Lee C. R., Lee J. W., Park S. W. & Park N. G. 6.5% efficient perovskite quantum-dot-sensitized solar cell. Nanoscale 3, 4088–4093 (2011). 21897986 10.1039/c1nr10867k

[b3] Kim H. S. *et al.* Lead iodide perovskite sensitized all-solid-state submicron thin film mesoscopic solar cell with efficiency exceeding 9%. Sci. Rep. 2, 591, doi: 10.1038/srep00591 (2012). 22912919 PMC3423636

[b4] Liu M. Z., Johnston M. B. & Snaith H. J. Efficient planar heterojunction perovskite solar cells by vapour deposition. Nature 501, 395–398 (2013). 24025775 10.1038/nature12509

[b5] Jeon N. J. *et al.* Solvent engineering for high-performance inorganic–organic hybrid perovskite solar cells. Nature materials 13, 897–903 (2014). 24997740 10.1038/nmat4014

[b6] Zhou H. P. *et al.* Interface engineering of highly efficient perovskite solar cells. Science 345,542–546 (2014). 25082698 10.1126/science.1254050

[b7] Best Research cells efficiencies. http://www.nrel.gov/ncpv/images/efficiency_chart.jpg, Date of access: 30/12/2014.

[b8] Saidaminov M. I. *et al.* High-quality bulk Hybrid perovskite single crystals within minutes by inverse temperature crystallization. Nat. Commun. 6, 7586, doi: 10.1038/ncomms8586 (2015). 26145157 PMC4544059

[b9] Dong Q. F. *et al.* Electron-hole diffusion lengths >175 um in solution-grown CH3NH3PbI3 single crystals. Science 347, 967–970 (2015). 25636799 10.1126/science.aaa5760

[b10] Dou L. T. *et al.* Atomically thin two-dimenstional organic-inorganin hybrid perovskites. Science 349, 1518- 1521 (2015). 26404831 10.1126/science.aac7660

[b11] Liu Y. C. *et al.* Two-Inch-Sized perovskite CH_3_NH_3_PbX_3_ (X = Cl, Br, I) crystals: growth and characterization. Adv. Mater. 27, 5176–5183 (2015). 26247401 10.1002/adma.201502597

[b12] Wong A. B. *et al.* Growth and anion exchange conversion of CH_3_NH_3_PbX_3_ nanorod arrays for light-emitting diodes. Nano Lett. 15, 5519–5524 (2015). 26192740 10.1021/acs.nanolett.5b02082

[b13] GRÄTZEL M. & Park N. G. Organometal halide perovskite photovoltaics: A diamond in the rough. Nano 09, 1440002, doi: 10.1142/S1793292014400025 (2014).

[b14] Niu G. D., Guo X. D. & Wang L. D. Review of recent progress in chemical stability of perovskite solar cells. J. Mater. Chem. A 3, 8970–8980 (2015).

[b15] Dong X. *et al.* Improvement of the humidity stability of organic–inorganic perovskite solar cells using ultrathin Al_2_O_3_ layers prepared by atomic layer deposition. J. Mater. Chem. A 3, 5360–5367 (2015).

[b16] Niu G. D. *et al.* Study on the stability of CH_3_NH_3_PbI_3_ films and the effect of post-modification by aluminum oxide in all-solid-state hybrid solar cells. J. Mater. Chem. A 2, 705–710 (2014).

[b17] Frost J. M. *et al.* Atomistic origins of high-performance in hybrid halide perovskite solar cells. Nano Lett. 14, 2584–2590 (2014). 24684284 10.1021/nl500390f PMC4022647

[b18] Yang J. L., Siempelkamp B. D., Liu D. Y. & Kelly T. L. Investigation of CH_3_NH_3_PbI_3_ degradation rates and mechanisms in controlled humidity environments using *in situ* techniques. ACS Nano 9, 1955–1963 (2015). 25635696 10.1021/nn506864k

[b19] Christians J. A., Miranda Herrera P. A. & Kamat P. V. Transformation of The Excited State and Photovoltaic Efficiency of CH_3_NH_3_PbI_3_ Perovskite upon Controlled Exposure to Humidified Air. J. Am. Chem. Soc. 137, 1530–1538 (2015). 25590693 10.1021/ja511132a

[b20] Leguy A. M. A. *et al.* Reversible Hydration of CH_3_NH_3_PbI_3_ in films, Single Crystals and Solar Cells. Chem. Mater. 27, 3397–3407 (2015).

[b21] Ito S., Tanaka S., Manabe K. & Nishino H. Effects of surface blocking layer of Sb_2_S_3_ on nanocrystalline TiO_2_ for CH_3_NH_3_PbI_3_ perovskite solar cells. J. Phys. Chem. C 118, 16995–17000 (2014).

[b22] Chen X. B. & Mao S. S. Titanium dioxide nanomaterials: synthesis, properties, modifications and applications. Chem. Rev. 107, 2891–2959 (2007). 17590053 10.1021/cr0500535

[b23] Mali S. S., Shim C. S. & Hong C. K. Highly porous zinc stannate(Zn_2_SnO_4_) nanofibers scaffold photoelectrodes for efficient methyl ammonium halide perovskite solar cells. Sci. Rep. 5, 11424, doi: 10.1038/srep11424 (2015). 26094863 PMC4476148

[b24] Bera A. *et al.* Fast crystallization and improved stability of perovskite solar cells with Zn_2_SnO_4_ electron transporting layer: interface matters. ACS Appl. Mater. Interfaces 7, 28404–28411 (2015). 26633572 10.1021/acsami.5b09182

[b25] Mali S. S. *et al.* Ultrathin atomic layer deposited TiO_2_ for surface passivation of hydrothermally grown 1D TiO_2_ nanorod arrays for efficient solid-state perovskite solar cells. Chem. Mater. 17, 1541–1551 (2015).

[b26] You J. B. *et al.* Improved air stability of perovskite solar cells via solution-processed metal oxide transport layers. Nature Nanotech. 11, 75–81(2016). 10.1038/nnano.2015.230 26457966

[b27] Cai B., Xing Y. D., Yang Z., Zhang W. H. & Qiu J. S. High performance hybrid solar cells sensitized by organolead halide perovskites. Energy Environ. Sci. 6, 1480–1485 (2013).

